# Investigating the interfaces of the epiphyseal plate: An integrated approach of histochemistry, microtomography and SEM

**DOI:** 10.1111/joa.13924

**Published:** 2023-06-30

**Authors:** Piero Antonio Zecca, Marcella Reguzzoni, Marina Borgese, Marina Protasoni, Marta Filibian, Mario Raspanti

**Affiliations:** ^1^ Department of Medicine & Surgery Insubria University Varese Italy; ^2^ Centro Grandi Strumenti University of Pavia Pavia Italy; ^3^ Istituto Nazionale di Fisica Nucleare, Pavia Unit Pavia Italy

**Keywords:** electron microscopy, growth plate, microtomography, ultrastructure

## Abstract

We investigated the interfaces of the epiphyseal plate with over‐ and underlying bone segments using an integrated approach of histochemistry, microtomography and scanning electron microscopy (SEM) to overcome the inherent limitations of sections‐based techniques. Microtomography was able to provide an unobstructed, frontal view of large expanses of the two bone surfaces facing the growth plate, while SEM observation after removal of the soft matrix granted an equally unhindered access with a higher resolution. The two interfaces appeared widely dissimilar. On the diaphyseal side the hypertrophic chondrocytes were arranged in tall columns packed in a sort of compact palisade; the interposed extracellular matrix was actively calcifying into a thick mineralized crust growing towards the epiphysis. Behind the mineralization front, histochemical data revealed a number of surviving cartilage islets which were being slowly remodelled into bone. In contrast, the epiphyseal side of the cartilage consisted of a relatively quiescent reserve zone whose mineralization was marginal in amount and discontinuous in extension; the epiphyseal bone consisted of a loose trabecular meshwork, with ample vascular spaces opening directly into the non‐mineralized cartilage. On both sides the calcification process took place through the formation of spheroidal bodies 1–2 μm wide which gradually grew by apposition and coalesced into a solid mass, in a way distinctly different from that of bone and other calcified tissues.

## Graphical abstract

This 3D rendering of a high‐resolution microCT illustrates the differences between the two boundaries of the epiphyseal plate. On the diaphyseal side (below) the cartilage interposed among hypertrophic chondrocytes undergoes an extensive mineralization, while on the opposite side its mineralization is marginal and large spaces of the cancellous bone open directly onto the soft cartilage.

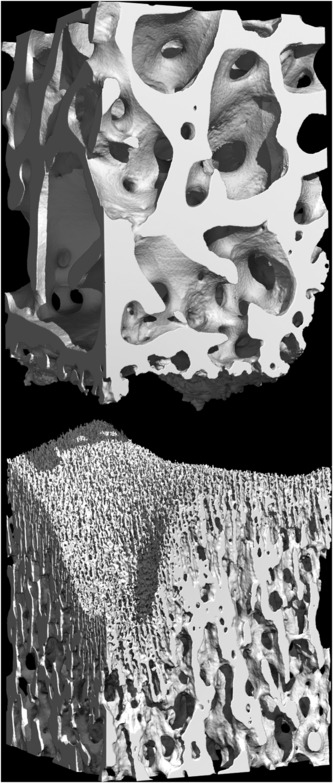

## 1 INTRODUCTION

The epiphyseal plate (or growth plate) essentially consists of a layer of cartilage interposed between two bony segments near the extremities of the long bones. It is the main actor in the coordinate elongation of bones, and this tissue and its functional modulation have been the subject of extensive research (Ballock & O'Keefe, 2003; Kozhemyakina et al., 2015; Marino, 2011; Xian, 2014).

From a structural standpoint this tissue, as other cartilages, consists of stiff fibrils immersed in a highly hydrated matrix. The cartilage fibrils are molecular alloys of collagen types II, IX, XI and XVI (Kassner et al., 2003; Mendler et al., 1989); other collagen types, especially types I and III, may be present (Aigner et al., 1993) but their function is still unclear (Styczynska‐Soczka et al., 2021; Wang et al., 2020). The interfibrillar matrix is composed of hyaluronic acid and of a variable population of glycoconjugates (Iozzo & Schaefer, 2015; Karamanos et al., 2021), mostly large, highly hydrated proteoglycans such as aggrecan and versican and small leucine‐rich proteoglycans bound to the collagen fibrils.

The extracellular matrix (ECM) is secreted by a resident population of chondrocytes, which remain dispersed in a relatively stiff ECM. These cells must remain alive and responsive even in a severely hypoxic context where the oxygen tension may be as low as 1% (Grimshaw & Mason, 2000). As a consequence, they have a rather low metabolic rate and almost no turnover.

The growth plate is distinctively asymmetrical (Anderson, 1969). Near the epiphysis there is a reserve zone whose chondrocytes are sparse and rounded; moving towards the diaphysis they become larger and flatter, begin to organize themselves in columns and form a proliferating zone. Farther on some chondrocytes reach a pre‐hypertrophic state and synthesize the signal protein Indian hedgehog (Ihh). This latter is part of a feedback loop involving the parathyroid hormone‐related peptide (PTHrP) and TGF‐ß, that keeps the chondrocytes in a proliferative state (Ballock & O'Keefe, 2003; Kozhemyakina et al., 2015; Kronenberg, 2003) by delaying their further differentiation. Eventually the chondrocytes enter a hypertrophic state (although they seem to be really swollen with water, rather than hypertrophic) and begin to express collagen type X (Lammi et al., 2002; Shen, 2005), while the intervening matrix calcifies. Whether the chondrocytes at this point undergo apoptosis or trans‐differentiation into bone lineage cells is still debated (Pazzaglia et al., 2020; Salucci et al., 2022; Wang et al., 2022).

Much less attention has been paid to the bony interfaces of this tissue. They are impossible to image with the sectional techniques typical of light microscopy (LM) and/or transmission electron microscopy (TEM), because these dictate a previous decalcification of the specimen that destroys the very object of their study; moreover, in radial sections the surface separating the two tissues appears just as a one‐dimensional line. In contrast, more recent techniques such as microCT and as scanning electron microscopy (SEM) after thermal removal of the soft matrix (Pazzaglia et al., 2016; Raspanti et al., 1994) can provide a face‐on visualization of the mineralization front and give access to new data.

The present research will focus on the morphology and on the functional aspects of the mineralization processes of bovine epiphyseal plate, complementing histological techniques with microCT and SEM.

## 2 MATERIALS AND METHODS

The epiphyseal plate was harvested at the slaughterhouse from the femoral head of calves of both sexes, aged 8–12 months. The head of the femur was dissected at the anatomical neck, split in half and immediately immersed in the fixative. The halves were then carefully divided into smaller fragments and processed as follows.

### 2.1 Light microscopy

Samples intended for light microscopy were fixed overnight in Karnowski's solution (5% paraformaldehyde +4% glutaraldehyde in 0. 1 M phosphate buffer, pH 7. 4), decalcified for 21 days in 2.5% hydrochloric acid plus 2. 5% acetic acid and embedded in paraffin. Sections were stained with haematoxylin–eosin, Stains‐All and Fast green and Safranin O, dehydrated in graded ethanol, cleared in xylene and mounted with Eukitt (Sigma‐Aldrich). All the samples were observed with a Nikon Eclipse 300 photomicroscope (Nikon) fitted with a Nikon Digital Sight DS‐U1 camera.

### 2.2 Scanning electron microscopy

Fresh specimens were frozen in solidifying propane at approximately −188°C and fractured under liquid nitrogen at −196°C in order to obtain clean, untouched surfaces. The specimens were then thawed in Karnowski solution and dehydrated in graded ethanol and hexamethyldisilazane.

Other fragments were briefly washed in 0.1 M phosphate buffer, thermally treated at 400°C for 24 h to remove the cells and the soft matrix and allowed to slowly return to room temperature.

All specimens were mounted on appropriate stubs with a colloidal silver glue, coated with 10 nm gold–palladium in an Emitech K550 sputter‐coater (Emitech) and observed with a FEI XL‐30 FEG high‐resolution SEM (now Thermo Fisher). Images were directly obtained as 8bpp, 1424 × 968 TIFF files.

### 2.3 MicroCT

Specimens were trimmed to approximately 3 × 5 × 10 mm, briefly fixed in Karnowski's solution and dehydrated in graded ethanol and hexamethyldisilazane as above. MicroCT measurements were performed at a nominal pixel size of 3 μm by means of a SkyScan 1276 scanner (Skyscan) setting a voltage–current combination of 55 kV–72 μA for the x‐ray source and applying a 0.25 mm aluminium filter. The images were captured every 0.18° through 180° of rotation without camera pixel binning, averaging four frames. The Z‐stack reconstruction was carried out with the SkyScan NRecon software, using the InstaRecon reconstruction engine, activating a Gaussian Smoothing Kernel, ring artefact reduction and beam hardening correction. Z‐stacks were converted into STL files using Mimics 21.0 (Materialise BV) and interactively rendered in 3D using Maya 2021 (Autodesk).

## 3 RESULTS

**FIGURE 1:**
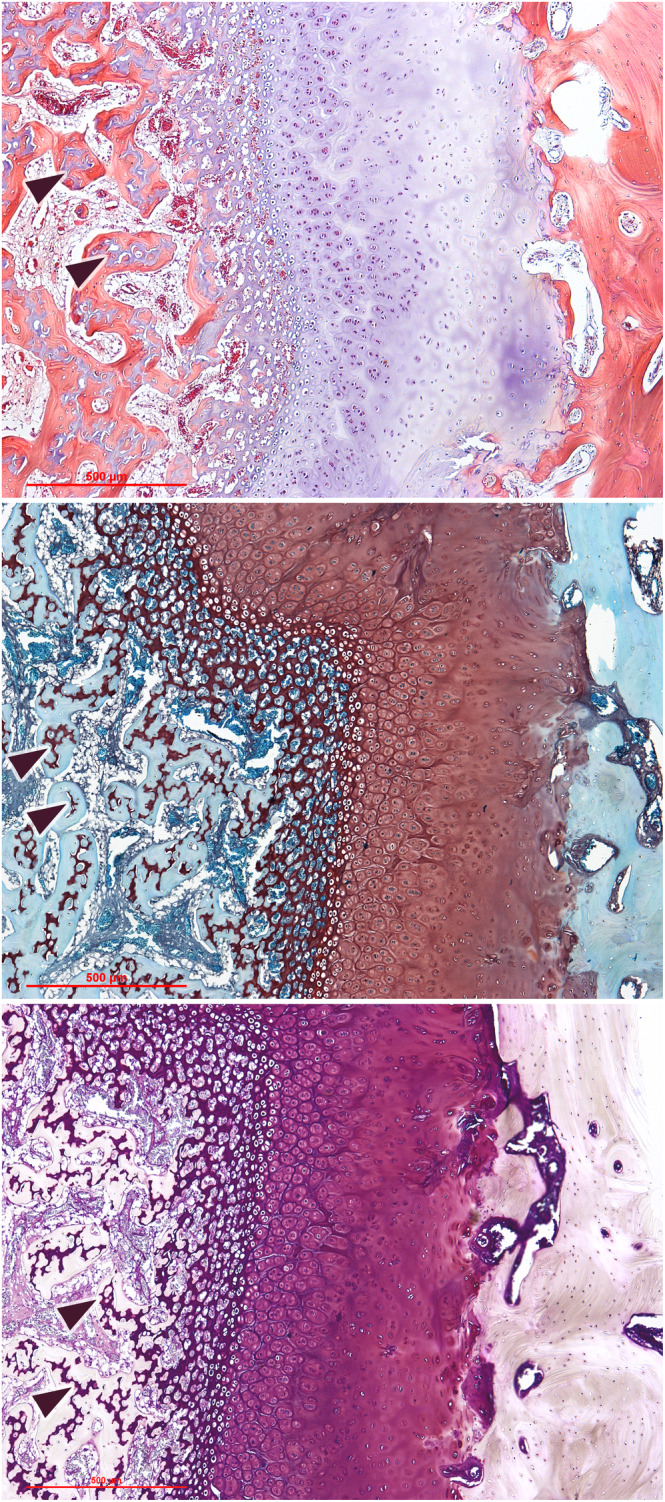
Histological cross‐sections of the epiphyseal plate, stained with haematoxylin–eosin (above), Safranin O–Fast green (middle) and Stains‐All (below). Each picture shows, from left to right, the diaphyseal bone, the hypertrophic cartilage, the proliferative and reserve zone and the epiphyseal bone. The diaphyseal bone always contains islets of cartilage still to be remodelled (arrowheads), their colour contrasting with the surrounding bone.

Independently from the staining used, histological sections (Figure 1) always showed the gradual transformation of the chondrocytes from the reserve zone through a proliferating stage and eventually into a hypertrophic condition. This transition proceeds in parallel with a progressive columnar organization of the cells. The bony segments facing the growth plate are widely dissimilar: the epiphyseal bone appears as a mature cancellous bone with wide interconnected vascular spaces, while on the diaphyseal side we can observe a gradual transition of the mineralized matrix deposed among the chondrocyte columns into a delicate and intricate trabecular bone, with thinner trabeculae and narrower spaces than the epiphyseal bone. All the stains we used showed a clear discrimination between bone and cartilage, and all consistently showed that numerous small islets of cartilage persist, intermingled with the newformed bone, well beneath the hypertrophic zone.

**FIGURE 2:**
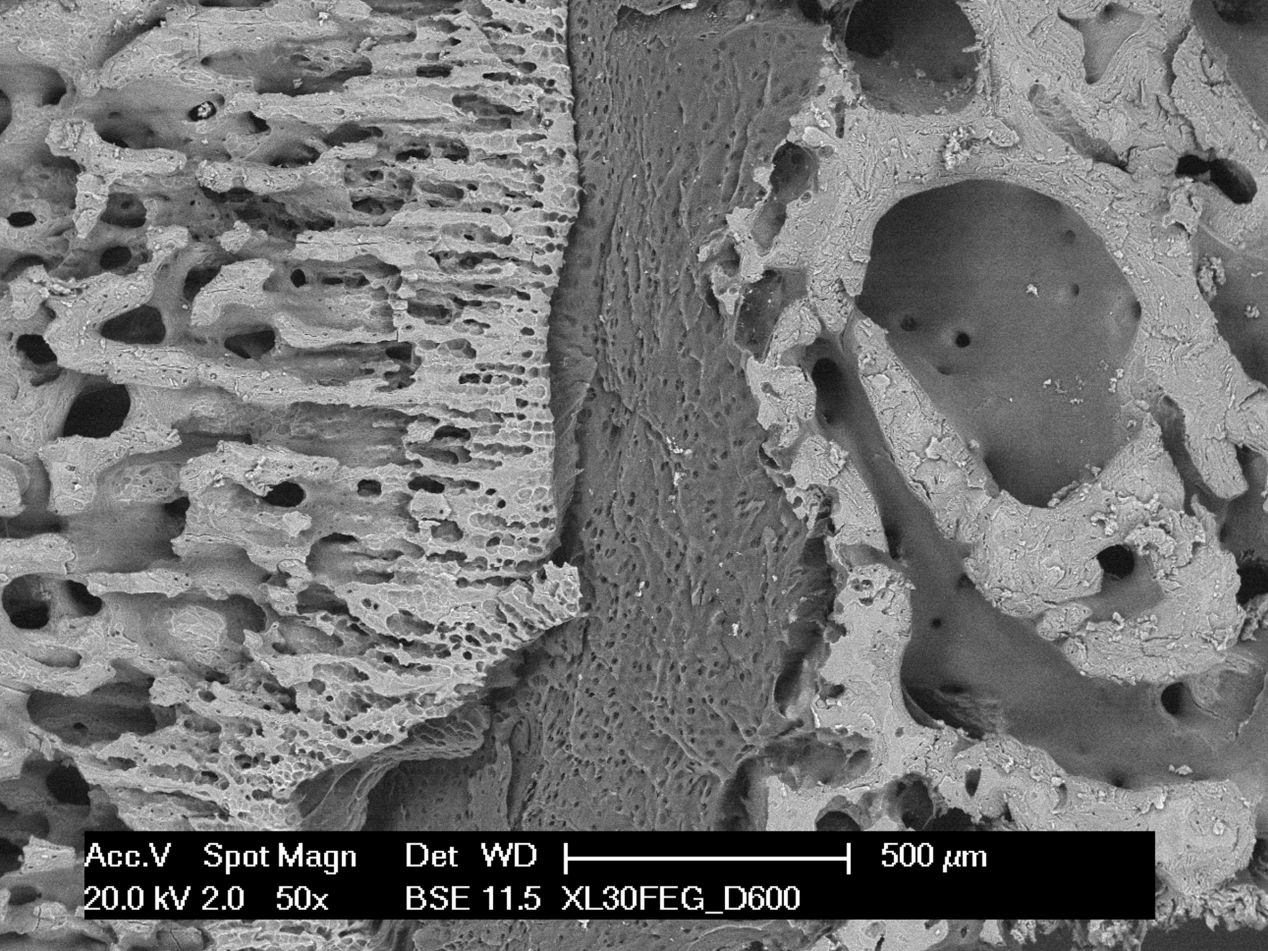
SEM micrograph, with approximately the same magnification and orientation of Figure 1. The backscattered electron imaging highlights the calcified portions, brighter than the soft matrix, and reveals the different texture of the two calcified compartments.

Since light microscopy requires the specimen to be decalcified, this technique cannot discriminate between the mineralized and unmineralized portions of these tissues. This is instead possible with scanning electron microscopy with backscattered electron imaging (Figure 2), and this approach clearly revealed the position of the mineralization front and the extension of the calcification process. In contrast with the sparse trabecular bone of the epiphyseal bone, the diaphyseal interface appears as a solid mineralized mass with a continuous, although rough, surface facing the hypertrophic cartilage.

**FIGURE 3:**
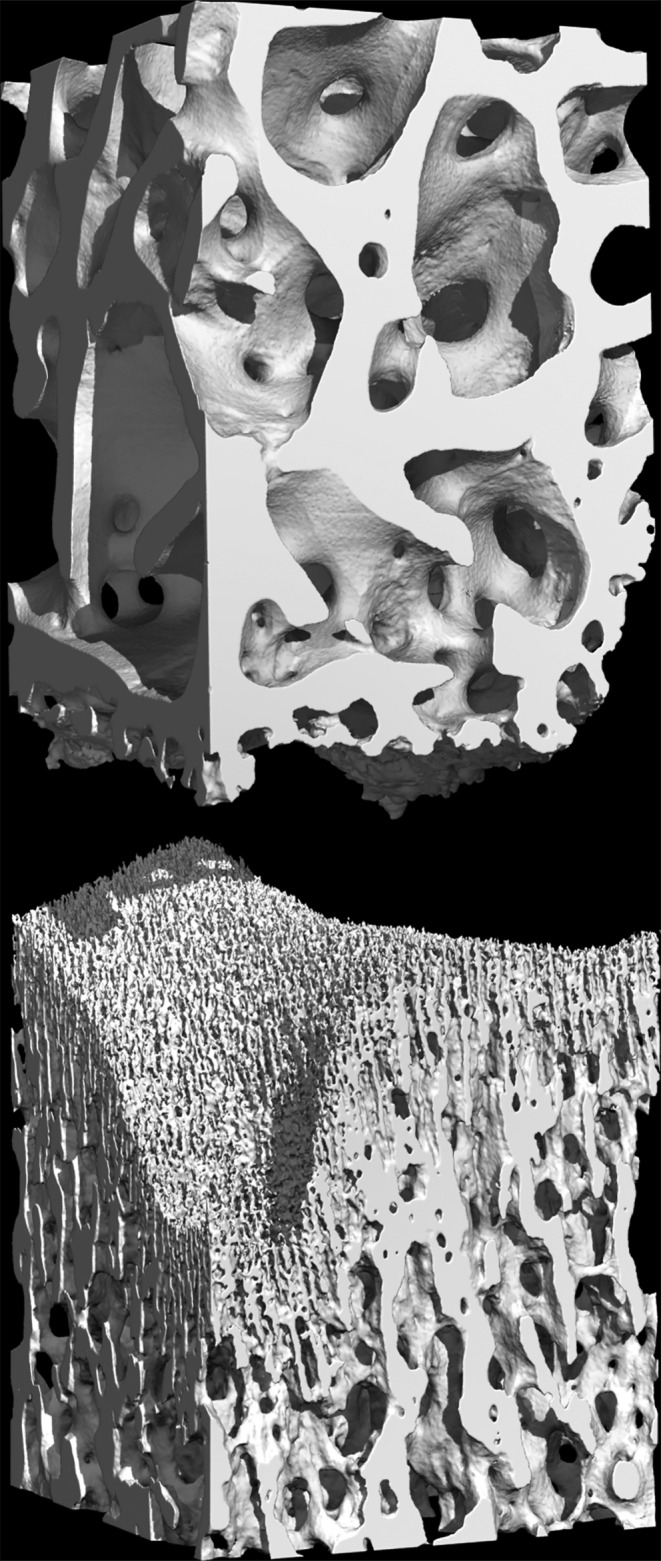
Graphic rendering of a microCT volume of 2 × 2 × 6 mm across the epiphyseal plate. This technique shows the two bony segments in their correct position; together they suggest the shape and volume of the interposed cartilage, which is not visible. This model has a lateral resolution of 6 μm and consists of about 23 million polygons.

**FIGURE 4:**
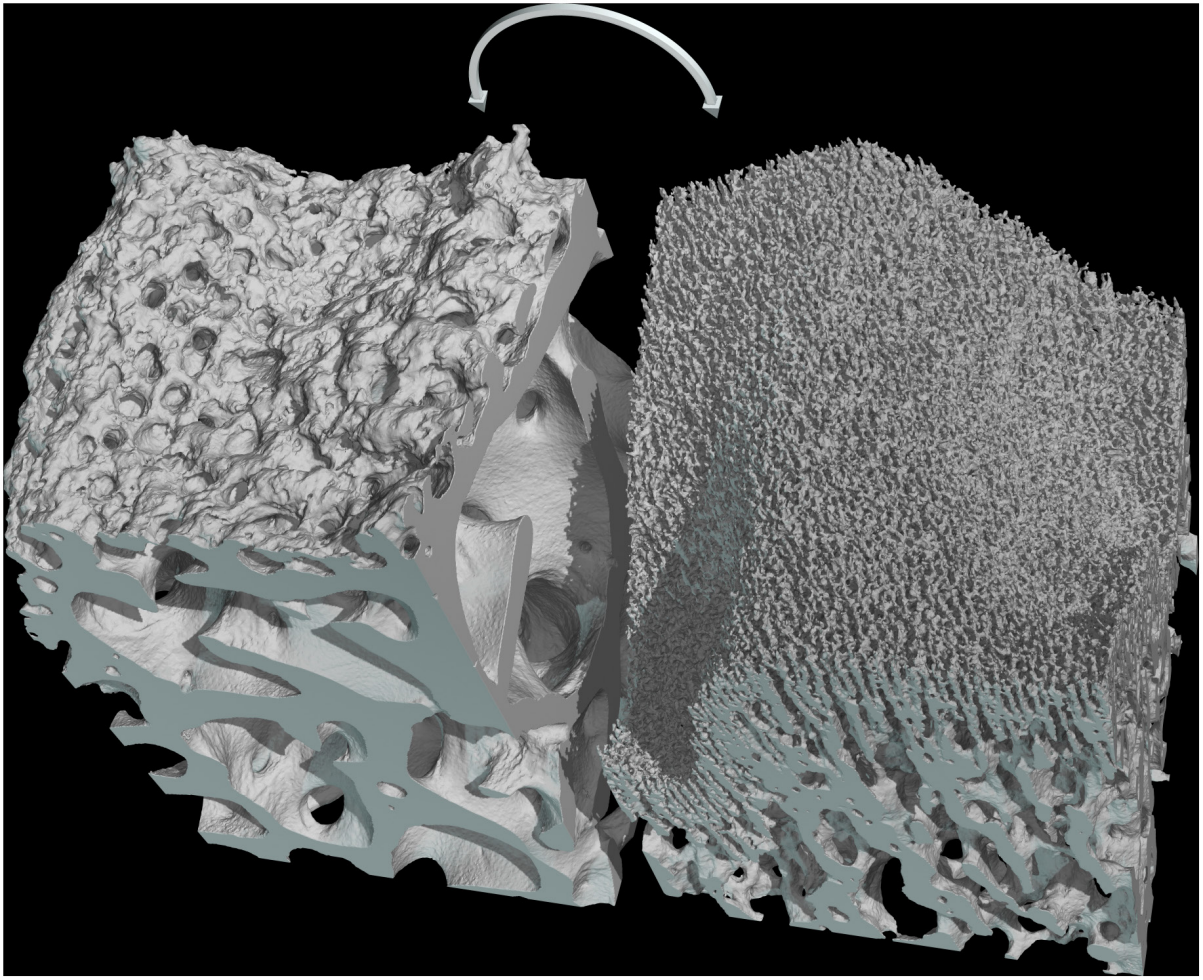
The same dataset, virtually split in half as indicated by the arrow in order to highlight the two bony interfaces. As in the previous picture, the openings of the epiphyseal bone (left) are clearly continuous with the vascular spaces of the underlying bone.

These aspects were confirmed and extended by the microCT and the interactive 3D imaging, which depicted a relatively large volume of the bone segments enclosing the epiphyseal plate (Figure 3) and which showed an equally large area of the two bony interfaces of the cartilage. This technique confirmed on a larger scale the wide differences between the two interfaces, with a compact columnar arrangement of hypertrophic chondrocytes being exclusively observable on the diaphyseal interface. The epiphyseal face, on the contrary, appeared more discontinuous and revealed a number of ample rounded apertures which reach the vascular spaces of the underlying trabecular bone (Figure 4).

**FIGURE 5:**
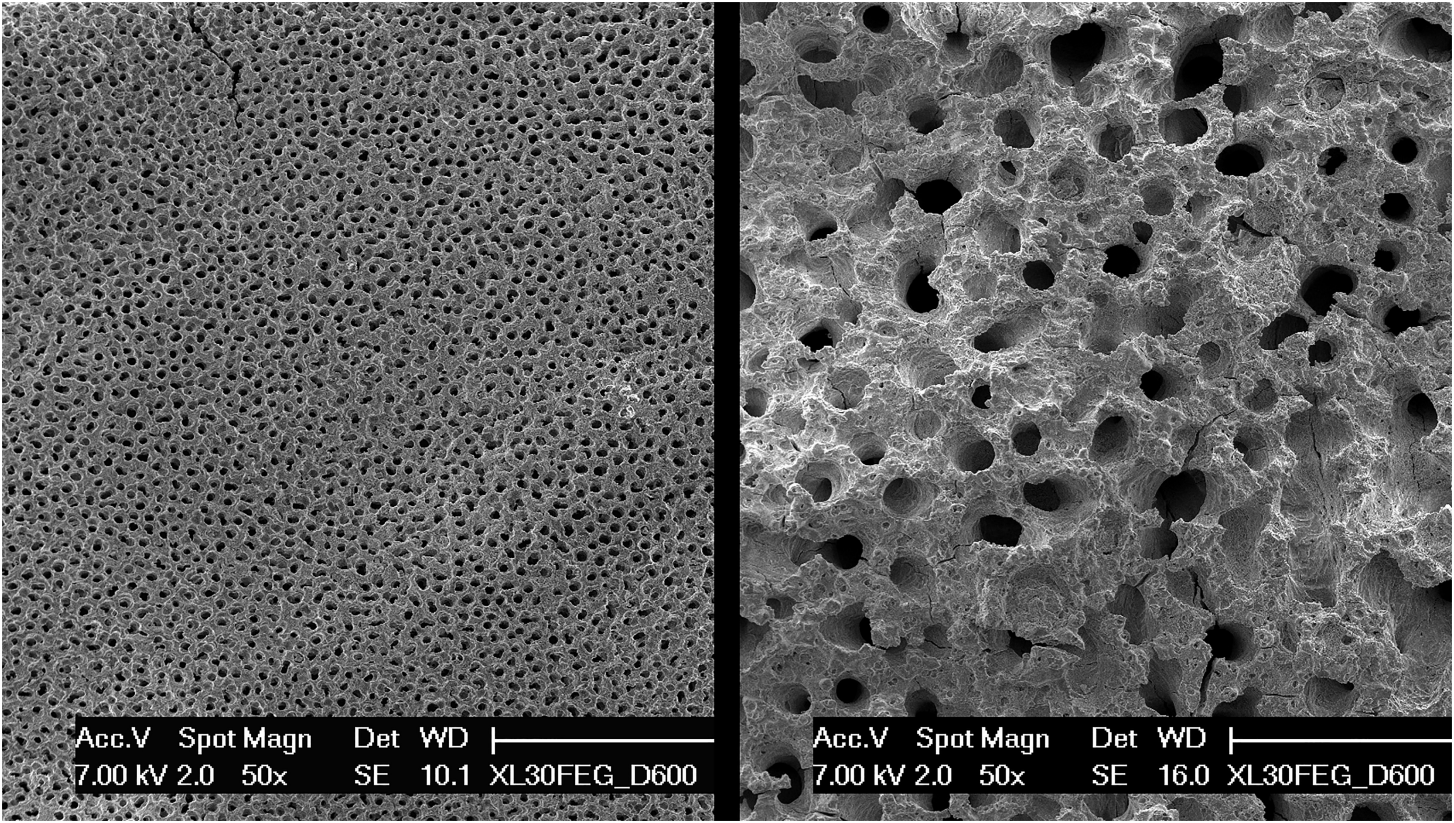
Two micrographs of a thermally deproteinated specimen confirm under the SEM the different aspects of the diaphyseal (left) and epiphyseal interfaces. Please note that the two images have the same magnification.

**FIGURE 6:**
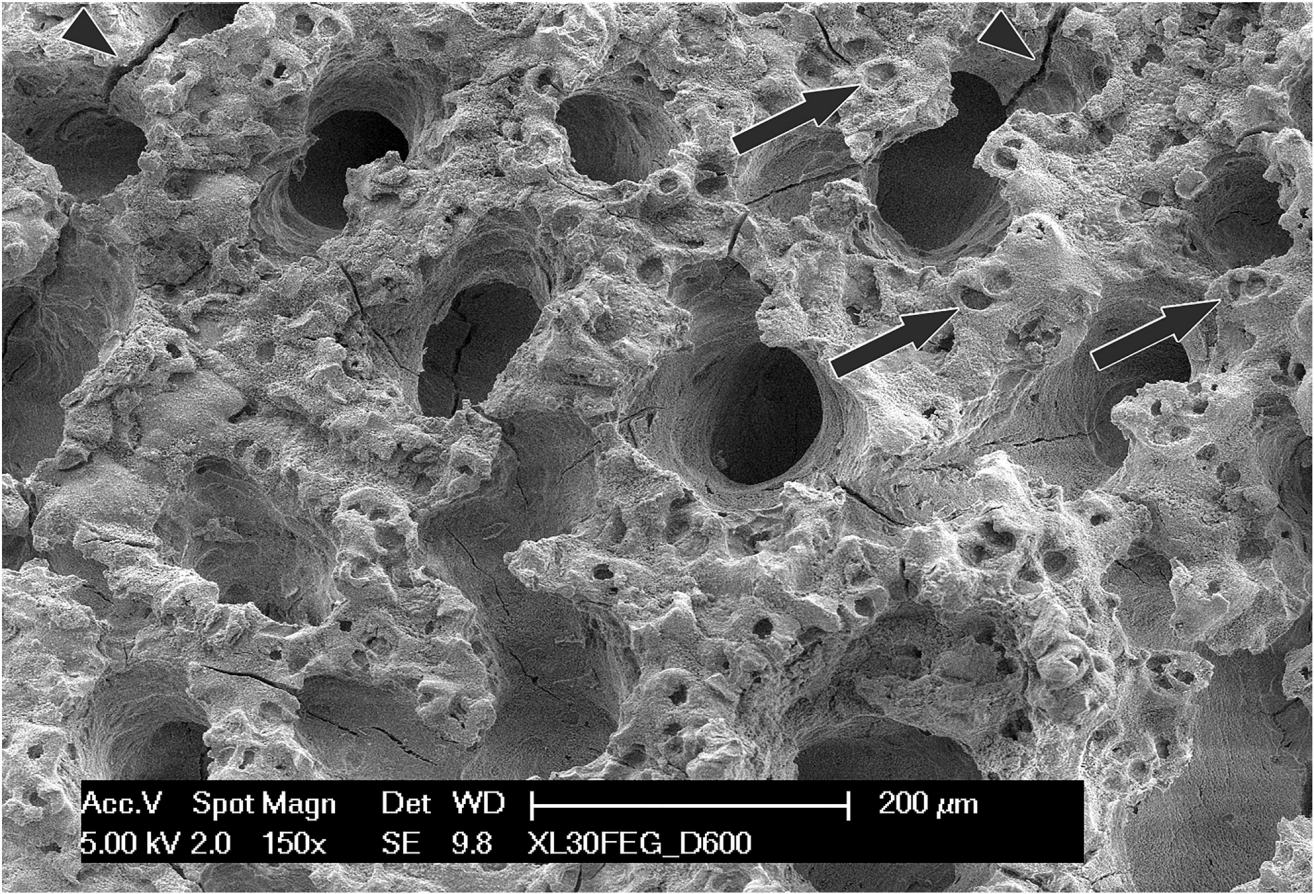
A higher magnification SEM micrograph of the epiphyseal face of a deproteinated specimen. The calcified cartilage is limited to the bridges among the large openings, with scarce and sparse chondrocyte cavities (arrows). This specimen shows several cracks (arrowheads) due to the heating/cooling stress.

Thermal treatment of the specimen vaporized the soft matrix without affecting the inorganic component, and allowed the scanning electron microscope to gain an unrestricted access to the mineralized surfaces (Figure 5). Consistently with the previous figure, a face‐on observation of the diaphyseal side revealed a continuous, rough plane pockmarked by the cavities left by the chondrocytes that lied halfway across the mineralization front. The epiphyseal interface, in contrast, showed wide, irregular, rounded cavities where the reserve zone of the cartilage could directly contact the vascular spaces of bone. A slightly higher magnification (Figure 6) was enough to reveal on the trabeculae of the epiphyseal interface the cavities left by the chondrocytes, that were scarce in number and rather sparse. In stark contrast with the diaphyseal side, where a thick layer of calcified cartilage gradually blends with the underlying bone, on the epiphysis the calcified cartilage only covers the bone with a partial and discontinuous coating.

**FIGURE 7:**
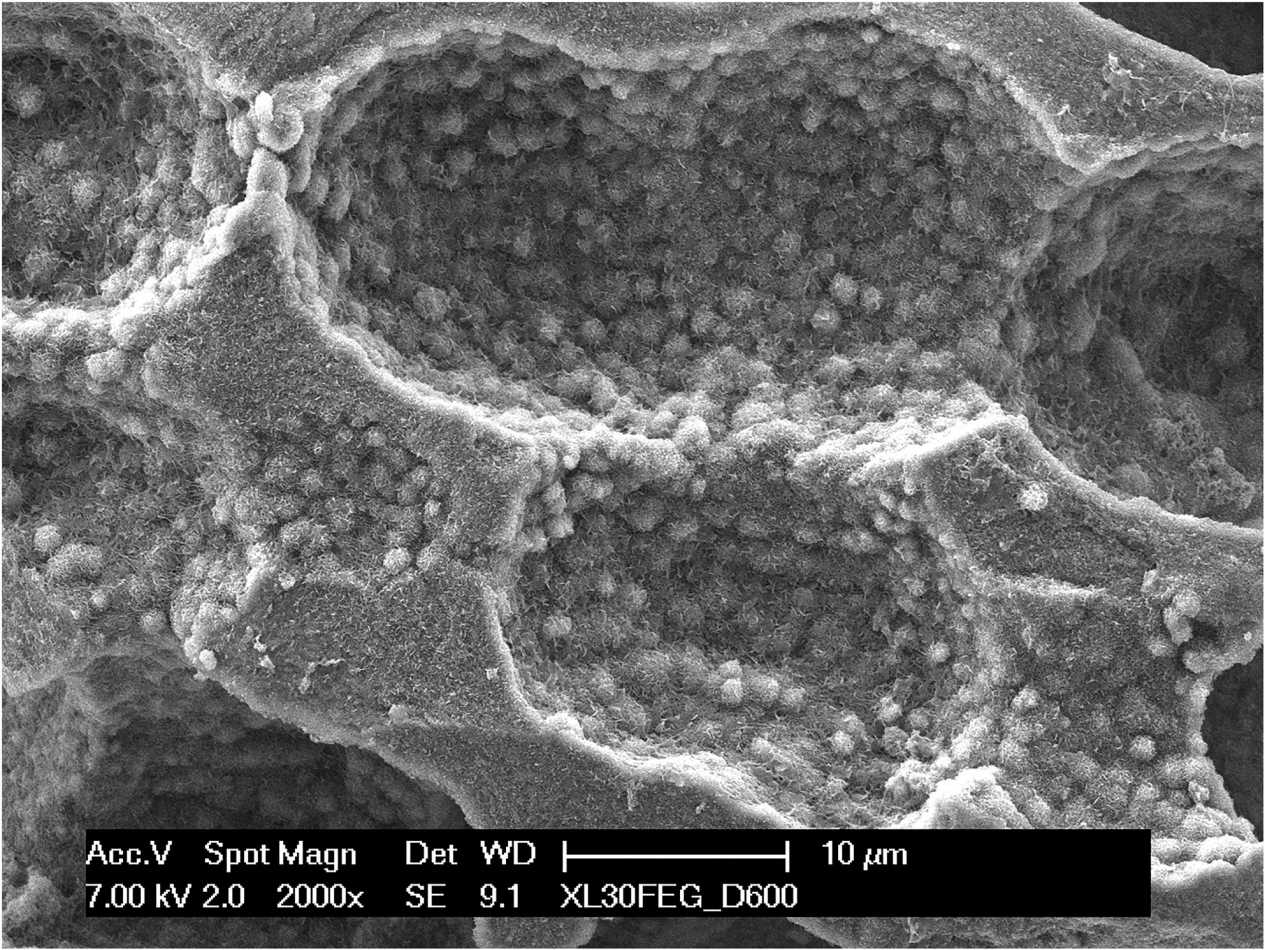
A detail of chondrocytic lacunae from a thermally deproteinated specimen. The visible surface, which represents the actual mineralization front, is entirely made of spheroidal inorganic particles.

**FIGURE 8:**
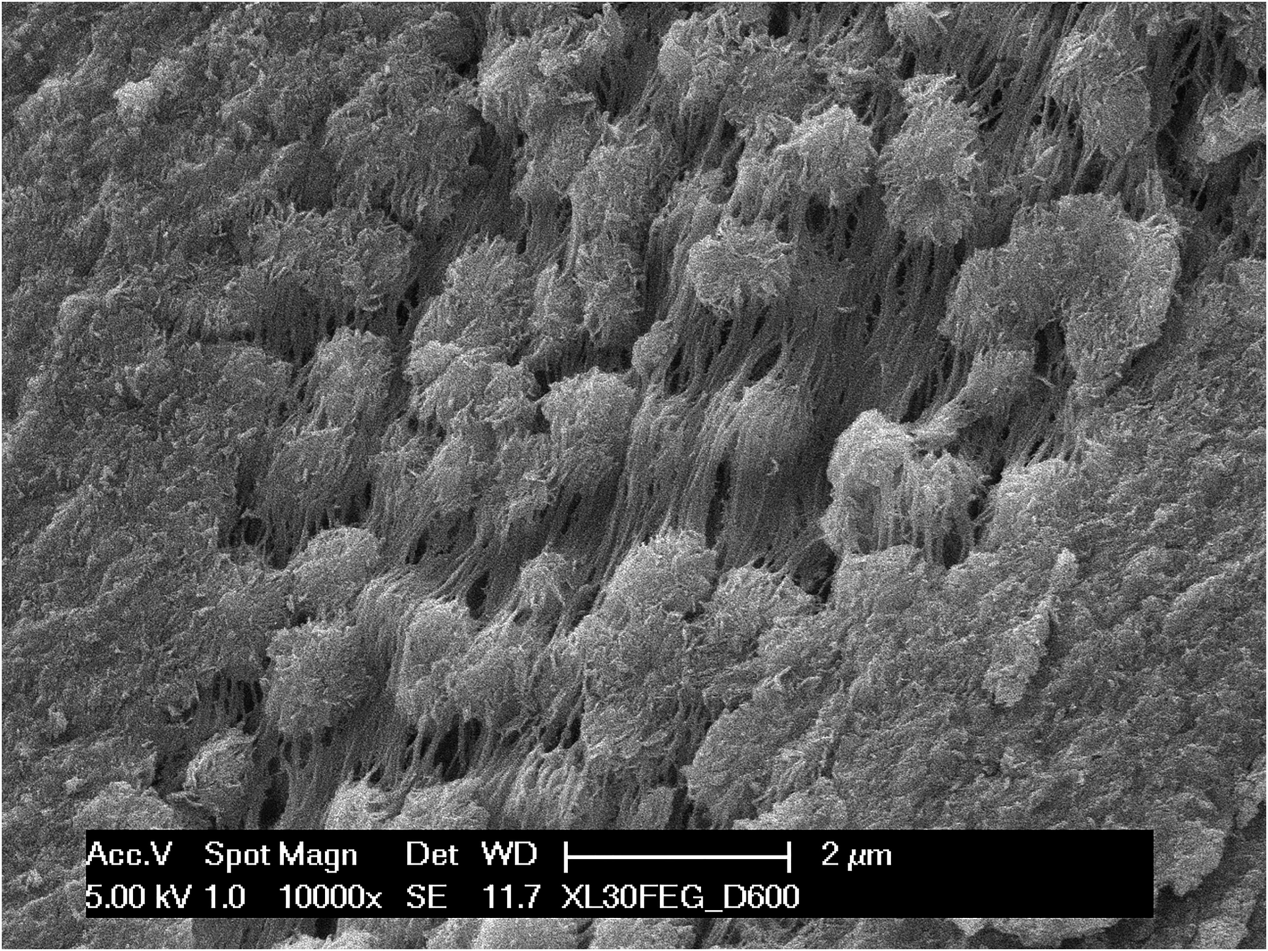
A higher magnification detail from an incompletely calcified zone of a native, untreated specimen. Small spheroidal particles appear interspersed among collagen fibrils.

**FIGURE 9:**
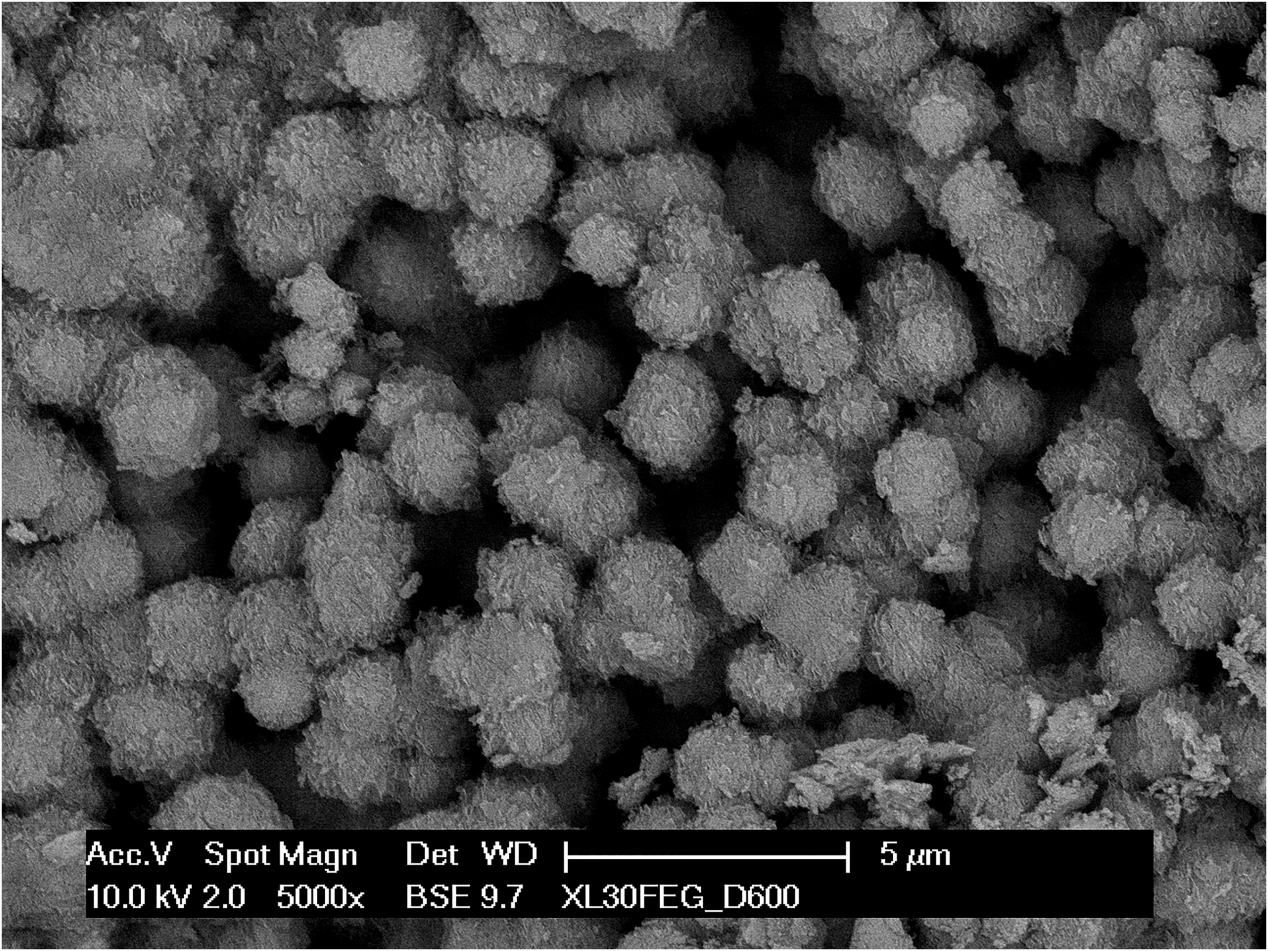
A detail of a similar specimen, imaged in backscattered electrons. This technique only shows the inorganic component, which is entirely limited to the spheroidal particles. No mineralized collagen fibrils are visible, at variance with what can be observed in other tissues.

**FIGURE 10:**
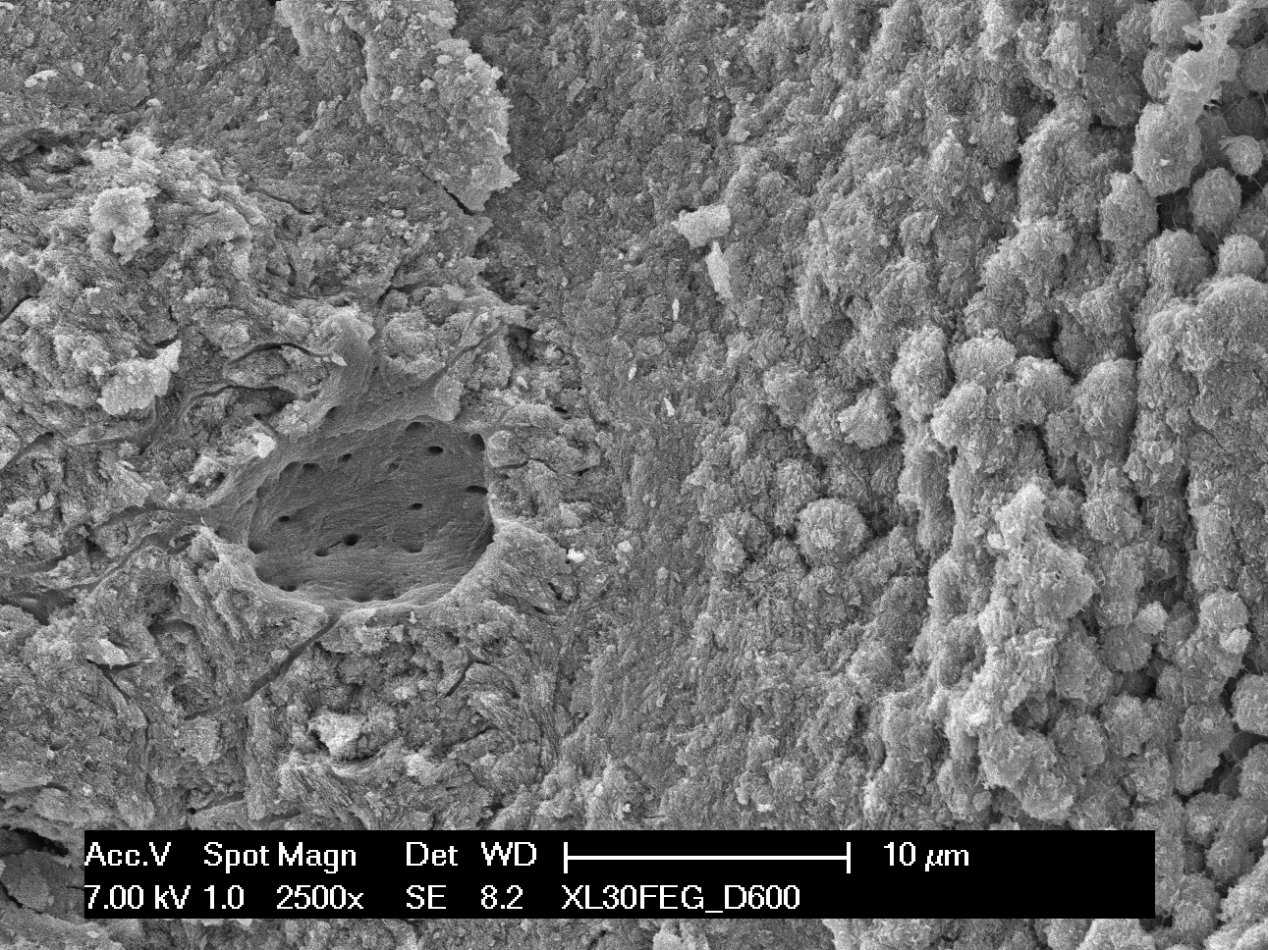
SEM micrograph of the calcified cartilage/bone interface. The picture highlights the contrast between the fibrous bone surrounding an osteocytic lacuna (left) and the distinctive spheroidal particles of the calcified cartilage (right) just a few micrometres away.

On both surfaces, a higher magnification of the chondrocyte cavities (Figure 7) revealed that the calcification of cartilage begins with the formation of spheroidal bodies in the range of 1–2 μm in diameter, which seem to form in the interstitial space (Figure 8) apparently with no direct mineralization of the collagen fibrils (Figure 9); subsequently they grow in number and size and eventually coalesce into a solid mass. They were more easily observable where the mineralization was still incomplete, that is, either on the lacunae surfaces, where the process begins, or vice versa in the locations more deeply buried into the calcified mass, where the process was slowed down by the limited supply of ions and energy that could seep through the already calcified matrix. Even in the mixed matrix beneath the diaphyseal mineralization front, where calcified cartilage and bone coexist (see Figure 1), at the ultrastructural level it was possible to discriminate the two tissues thanks to the dissimilarity between the fibrous texture of bone and the spheroidal aggregates of the partially mineralized cartilage (Figure 10).

## 4 DISCUSSION

We must above all point out that the mineralization front is often, and incorrectly, identified in literature with the thin darker line that remains visible in decalcified specimens and is defined as *tidemark*. But the tidemark is an inconstant finding and multiple tidemarks are often visible, while at any given moment the mineralization front is evidently unique; in the present study it was reliably revealed by microtomography and by backscattered electron imaging. On the other hand, these techniques cannot easily discriminate between bone and calcified cartilage, which can be instead easily identified by histochemical techniques as shown in Figure 1.

A first observation is that the resting cartilage layer facing the epiphysis undergoes just a marginal calcification, in stark contrast with the other side. This is consistent with the fact that mineral deposition is usually accompanied by secretion of collagen X (Lammi et al., 2002), which is distinctive of hypertrophic chondrocytes in the growth plate and in the articular cartilage (Shen, 2005). It must be noted that calcified cartilage is credited with mechanical parameters intermediate between soft cartilage and bone (Boyde, 2021; Mente & Lewis, 1994) and it seems to be essential for a stronger mechanical junction between the two tissues (Evans & Pitsillides, 2022), but on the epiphyseal interface the thickness of calcified cartilage here seems minimal and the cartilage–bone contact is limited to discontinuous, widely spaced trabeculae.

On the other hand, some of the vascular spaces of the epiphyseal bone impinge directly on the resting zone of cartilage with no intermediation, a thing that is bound to have important consequences on the metabolism of this tissue. The commonly accepted knowledge is indeed that the cartilage, being avascular, is fed by passive diffusion from the perichondrium; but in large animals (and in man) this can lie several centimetres away, too far for a viable diffusion. In the articular cartilage the main supply route is the synovial surface, and it is still debated whether there may be any contribution by the subchondral bone (Arkill & Winlove, 2008; Lepage et al., 2019; Oliveira Silva et al., 2020; Pan et al., 2009). The very low oxygen content of the deeper zones seems to suggest the contrary (Grimshaw & Mason, 2000). On the epiphyseal side of the growth plate oxygen and nutrients can flow unhindered from the vascular spaces to the adjoining matrix, a fact already observed in the articular cartilage (Lyons et al., 2006) and whose implications may have been overlooked in studies carried out on smaller animals (Delgado‐Martos et al., 2013; Fernández‐Iglesias et al., 2021). The epiphyseal bone–cartilage contact surface is very irregular (see Figure 4) and it is difficult to evaluate how much of this surface is represented by vascular spaces but it may be not far from 50%, and it is therefore plausible that the bone marrow, rather than perichondrium, represents an important supply of oxygen and nutrients to the growth plate, and from here to the elongating bone as well.

This implies that the metabolism of the growth plate must be very different from that of the articular cartilage, where an hypoxic state seems crucial in maintaining a chondrocytic phenotype (Hirao et al., 2006; Sieber et al., 2020) by promoting the synthesis of collagen types II and IX (Hansen et al., 2001) and of glycoconjugates (Bae et al., 2018), while an higher oxygen content would promote the synthesis of collagen types I and X (Sheehy et al., 2012).

Another observation is that the initial mineralization is consistently accompanied by the appearance of spheroidal mineral bodies, visible both on natural surfaces and on fracture planes. In places where the mineralization in still underway they are more easily identifiable, but they are present everywhere. They have been reported in other cartilages as well (Anderson, 1969; Claassen et al., 2017; Jaroszewicz et al., 2021) and represent a different mineralization pattern than the collagen‐driven mineralization of bone (Christoffersen & Landis, 1991). Briefly, in bone and in calcifying tendons the process begins simultaneously in a huge number of independent collagen fibrils: tiny, needle‐shaped mineral particles appear in the gap zone of type I‐based collagen fibrils and subsequently grow into plate‐like particles, invade the overlap zone and eventually swell and loosen the collagen fibril which merge with neighbouring ones (Landis et al., 1996; Reznikov et al., 2018; Xu et al., 2020). In contrast, in cartilage the mineral phase appears in the interfibrillar space in form of a limited number of globular aggregates, which subsequently grow until they coalesce with other adjoining particles. These globular bodies have been related to matrix vesicles acting as the seed of initial mineral deposition, although in this tissue they grow larger than is usually reported (Bottini et al., 2018).

Matrix vesicles have been described in several tissues as small, membrane‐bound bodies that arise by budding (or perhaps by secretion) by different cell types. In the extracellular space they bind to collagen fibrils. Several ion channels and enzymes are attached to their membrane, and exogenous inorganic ions are actively transferred and concentrated in the vesicle interior where the initial nucleation of mineral particles takes place (see Boyan et al., 2022 and Kirsch et al., 1997). Eventually these particles puncture the vesicle and grow into the interstitial space by superficial apposition forming distinctive fractal‐like globular particles (Christoffersen & Landis, 1991; Landis et al., 1992).

Our technical approach is unable to directly visualize the matrix vesicles, but what we observed is quite suggestive of their outcome. If they are actually involved in the process it seems consequential that, once the vesicle membrane are dissolved, the mineral particles have no relation anymore with the type II‐containing collagen fibrils. In calcified cartilage we never observed the mineral‐infiltrated collagen fibrils typical of bone, dentin and calcifying tendon, which would be easily recognizable with backscattered electron imaging (see Figure 9) and in deproteinated specimens. This suggests that collagen fibrils take no active part in the process but are just being displaced by the growing mineral spheroids. Their role, if any, is yet to be clarified.

The hypothesis that mineral spheroids form and grow within the extracellular matrix with no direct involvement of the collagen component, displacing an equal volume of water that in turn enters the chondrocytes, appears a reasonable starting point but at present is still conjectural. Further research is needed to clarify the ultrastructural and molecular details of cartilage mineralization.

## AUTHOR CONTRIBUTIONS

Piero Antonio Zecca was involved in concept, data acquisition and critical revision. Marina Borgese was involved in data acquisition. Marcella Reguzzoni and Marina Protasoni were involved in data acquisition and project management. Marta Filibian was involved in data acquisition and data analysis. Mario Raspanti was involved in Supervision and manuscript drafting.

## Data Availability

The data of this study are available from the corresponding author upon reasonable request.
